# On Phlebitis

**Published:** 1850-08

**Authors:** 


*On Phlebitis.*—By Dr. Novellis.—Dr. Novellis states that this disease has been very frequently observed by him in the military hospital at Alessandria; and indeed it so frequently follows venesection, as to lead the patients to manifest great reluctance to undergo this operation. He thinks the following circumstances he has observed while studying it there deserving of record.

1. It is most frequently observed in those patients who are placed in dark or damp abodes, humidity especially seeming to be a frequent cause of its production.

2. It appears especially when variola is prevalent in the hospital. Not, however, that it affects the variolous patients; but while variola appears, for example, in one ward, patients suffering from inflammatory diseases in the others are more liable to phlebitis, such liability diminishing again when the variola has disappeared.

3. On the other hand, typhus is never found prevalent when phlebitis is; an antagonism seeming to exist between the two.—*Omedei Annali*, vol. cxxiv, p. 206.

